# High risk of depression, anxiety, and an unfavorable complex comorbidity profile is associated with SLE: a nationwide patient-level study

**DOI:** 10.1186/s13075-022-02799-6

**Published:** 2022-05-19

**Authors:** Fruzsina Kósa, Péter Kunovszki, Judit Gimesi-Országh, Melinda Kedves, Melinda Szabó, Chetan S. Karyekar, György Nagy

**Affiliations:** 1 Janssen Global Services LLC, Budapest, Hungary; 2 Department of Rheumatology, Hospital of Bács-Kiskun County, Kecskemét, Hungary; 3 New Saint John Hospital and Outpatient Clinic, Budapest, Hungary; 4 Janssen Research & Development, Spring House, PA USA; 5 Department of Rheumatology and Clinical Immunology, Department of Internal Medicine and Oncology, Semmelweis University, Budapest, Hungary; 6 Heart and Vascular Center, Semmelweis University, Budapest, Hungary; 7 Department of Genetics, Cell- and Immunobiology, Semmelweis University, Budapest, Hungary

**Keywords:** Systemic lupus erythematosus, Hungary, comorbidities, Depression, Anxiety

## Abstract

**Objectives:**

The aim of this national population-based, retrospective database study is to compare the comorbidity profiles of systemic lupus erythematosus (SLE) patients and general population controls matched for age, gender, and region and assess the risk of depression or anxiety when controlled for age, gender and adjusted for the Charlson Comorbidity Index (CCI).

**Methods:**

Claims data of 1051 patients diagnosed with SLE (full population between January 01, 2011, and December 31, 2014) from the Hungarian National Health Insurance Fund have been analyzed against matched controls (1:5 ratio) with a follow-up of 30 months. The first record of SLE diagnosis was considered the diagnosis date. The odds ratio (OR) and 99.9% confidence interval (CI) of having depression or anxiety among patients with SLE vs. controls have been assessed using logistic regression models.

**Results:**

SLE patients report more comorbidities than the matched general population both in pre- and post-index periods (mean CCI 1.79 vs. 1.15 and 2.78 vs. 1.22 [both *p*<0.001], respectively). Both SLE patients and controls diagnosed with depression or anxiety had significantly higher CCI than those without comorbid depression or anxiety (*p*<0.001). However, SLE patients had a twofold higher risk of depression or anxiety than matched controls when controlled for age, gender, and adjusted for CCI.

**Conclusion:**

Our analysis indicates the enormity of comorbidity burden in SLE, especially that of anxiety and depression. The size and complexity of the comorbidity burden emphasizes the importance of early diagnosis and intervention with comprehensive modalities incorporating attention to comorbidities in SLE patients.

**Supplementary Information:**

The online version contains supplementary material available at 10.1186/s13075-022-02799-6.

## Key messages

- SLE patients show a 2.2-fold higher risk of depression or anxiety on controlling for age and gender and adjusting for the CCI.
- The complexity of the burden of the disease requires intervention with new and comprehensive treatment.
- Clinicians need to regularly evaluate SLE patients for symptoms of depression and anxiety.

## Introduction

Systemic lupus erythematosus (SLE) is a chronic, heterogeneous systemic autoimmune disease associated with multiple clinical complications and comorbidities, which may lead to significant organ damage resulting in increased disease burden and mortality [1–3]. Both multi-organ involvement and treatment for SLE may lead to a higher risk of comorbidities [4]. The prevalence of SLE in Europe has been estimated to be 25–91 per 100,000 [5] while recent evidence reported the prevalence of SLE in Hungary to be 70.5 per 100,000 [1].

The European Alliance of Associations for Rheumatology recommends prompt evaluation if comorbidities are suspected in a patient with SLE [6]. Commonly observed comorbidities in patients with SLE include cardiovascular diseases, infections, psychosis, depression, chronic pulmonary diseases, musculoskeletal diseases, neoplasms, and renal diseases [7–9]. Moreover, the incident risk of these comorbidities is greater in patients with SLE compared with the general population as reported by two population-based studies [7, 9].

SLE along with any presenting comorbidities may lead to pain [10], increased work disability [11] as well as increased financial strain [12] resulting in a lower socioeconomic status. Moreover, the latter has also been associated with increased SLE severity and poor outcomes [13, 14], resulting in a vicious cycle. It is well documented that the aforementioned factors are associated with anxiety and depression in patients with SLE [10, 15]; additionally, anxiety and depression themselves might be neuropsychiatric manifestations of SLE [16]. A recent systematic review and meta-analysis reported the pooled prevalence of depression and anxiety in patients with SLE to be 35.0% (95% confidence interval [CI] 29.9–40.3%) and 25.8% (95% CI 19.2–32.9%) [17]. In addition, depression and anxiety are more common in patients with SLE compared to the general population [18].

Comorbid depression and anxiety are associated with low health-related quality of life (HRQoL) and greater work disability and economic burden in patients with SLE [19–21]. Therefore, it is essential to understand the risk of these comorbidities in SLE to optimally manage the disease. However, there is limited evidence on the complex comorbidity profile of SLE. Hence, this current full populational, nationwide, retrospective study aims to compare the comorbidity profile of adult patients with SLE and controls matched for age, gender, and region and to assess if the risk of having depression and anxiety is different when adjusted for the Charlson Comorbidity Index (CCI) in addition to controlling for age and gender.

## Methods

### Data source

De-identified records were obtained from the longitudinal, nationwide database of the Hungarian National Health Insurance Fund (NHIF). This database contains detailed healthcare service records including patient-level demographic data as well as inpatient, outpatient, and prescription drug data for the whole population of Hungary (approximately 10 million people) and is linked by the social security number, a unique patient identifier that enables longitudinal patient pathway analysis.

The NHIF handles patient data based on law (*Act No. 80/1997 on mandatory health insurance* coverage) and data access is provided for real-world analyses (based on *Act 63/2012 on the re-use of public data*)*.* Only NHIF had direct access to patient-level data; other parties could only access the data indirectly through NHIF as per NHIF’s data privacy regulations. Due to this regulation, patient-level consent was not required for the analysis.

### Study population

**Fig. 1:**
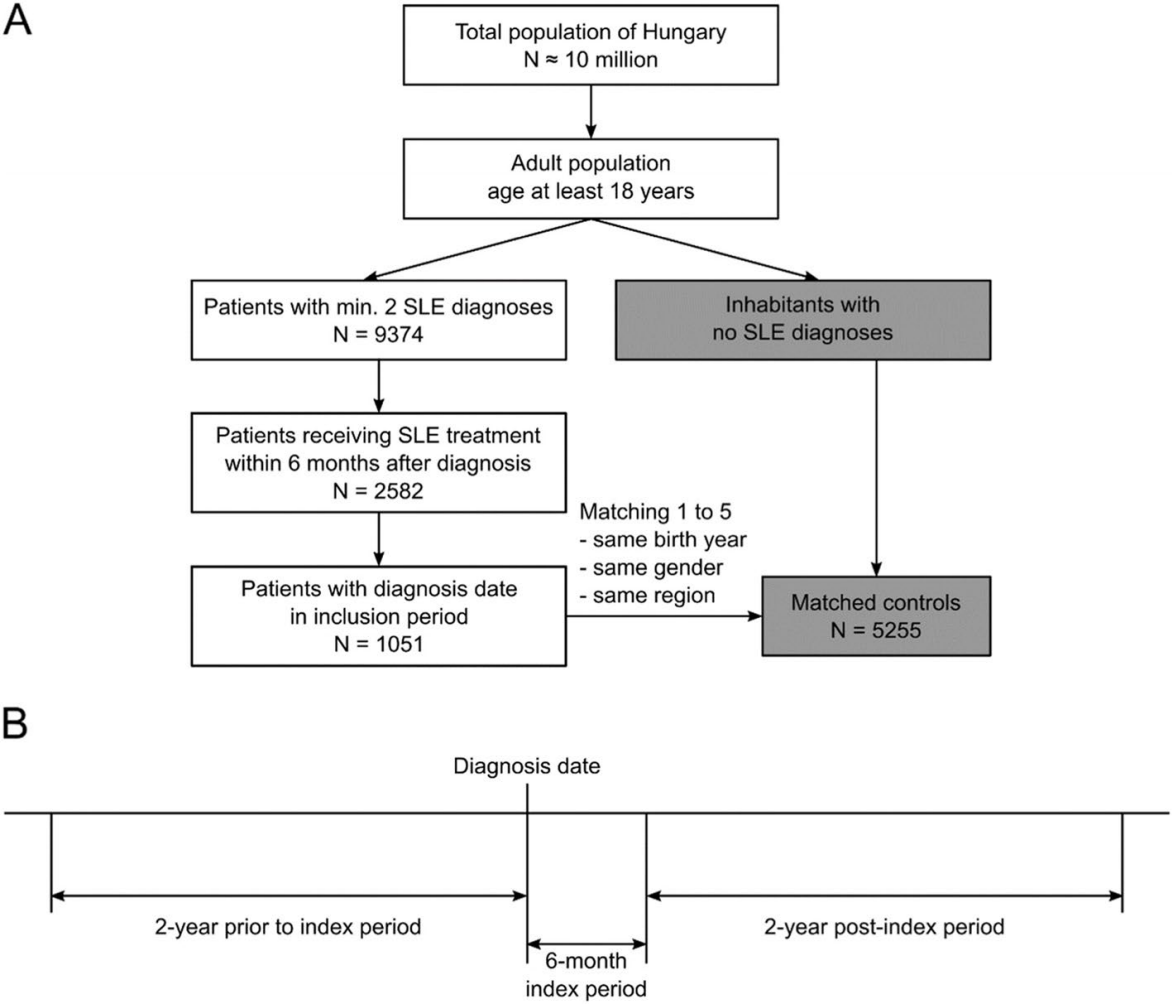
**A** Schematic of patient inclusion. **B** Patient timeline in relation to the diagnosis date. Shaded rectangles denote patients without an SLE diagnosis. Abbreviations: SLE, systemic lupus erythematosus

The study period ranged from January 01, 2009, to June 30, 2017. Patients who were diagnosed with SLE as per the International Classification of Diseases, 10th Revision (ICD-10) code M32* [22] during the inclusion period (January 01, 2011, to December 31, 2014), had no recorded SLE diagnosis for at least 2 years before the first record of SLE diagnosis in the inclusion period (diagnosis date), and had a record of relevant SLE therapy (Supplementary Table S1) within the 6 months from the diagnosis date (index period) (as per our prior research [1]) were included (Fig. 1A). Patients were followed up for at least 2 years after the index period (Fig. 1B). A cohort of reference individuals from the same database without any diagnosis of SLE (control group) was created matching on age, gender, and geographical region in a 1:5 ratio. The members of this control population had their index periods defined to be exactly the same as the index period of that SLE patient to whom they were matched. Comorbidities in the prior and post periods are in relation to this derived index period for controls.

Based on the average amount of prednisone-equivalent steroid dose prescribed in the 6-month index period, patients were classified into four categories (0 mg/day, ≤7.5 mg/day, ≤20 mg/day, and >20 mg/day).

### Ethics approval

The analysis was done based on the ethical approval of the Committee of Science and Research Ethics of the Medical Research Council of Hungary, approval number 53229-2/2018/EKU dated November 22, 2018.

### Outcomes

Patient population characteristics of interest included age, gender, comorbidities, age-adjusted CCI, and mortality. Comorbidities based on ICD-10 codes (Supplementary Table S2) were confirmed if the patient had at least one record with the comorbidity as the main diagnosis/main cause during inpatient care or had at least two records of the comorbidity on two different calendar days within 365 days of each other. Specifically, a patient was identified as having depression in the pre- or post-index period if they had any of the F31*, F32*, or F33* ICD-10 codes reported in such a way in the pre- or post-index period respectively. In case of anxiety, the ICD-10 code F41* was used. Comorbidities are presented only if there were 10 or more patients for the protection of individual patient data. The age-adjusted CCI was calculated based on the presence of comorbidities, for each comorbidity in scope, the patients had a score ranging between 1 and 6 and these scores were added together. Additional scores were obtained based on age. Supplementary Table S3 contains the comorbidities and the associated scores for the calculation of CCI [23].

The primary objective is to describe the complex comorbidity profile among patients with SLE and compare them with the matched general population, and the secondary objective is to estimate the role of SLE on the risk of having depression and anxiety.

### Statistical analysis

We calculated means and standard deviations for continuous variables and counts and percentages for categorical variables. In the Base Case Analysis, we assessed the odds ratio (OR) of presence of a wide range of different comorbidities post index period, including depression and anxiety among patients with SLE vs. controls and among SLE patients on different steroid doses vs. no steroid use (with 99.9% CI) using a logistic regression model adjusted for age and gender. We considered each comorbidity independently of each other in separate models. As we aimed for a family-wise error rate of 5%, the comparison-wise alpha was chosen to be 0.1% based on the Bonferroni correction. In the Adjusted Analysis, we applied the above model only for depression and anxiety, with the logistic regression model adjusted for the CCI as well as age and gender. Pre-index CCI is used in the adjustment, to enable the assessment of a cause-and-effect-type relationship. In administrative healthcare data the missing data cannot be discovered, if a diagnosis or intervention is not recorded then imputation is not possible. Therefore, missing data handling was not performed. We performed all the statistical tests using R software (R: A language and environment for statistical computing. R Foundation for Statistical Computing, Vienna, Austria. URL https://www.R-project.org/).

## Results

### Population characteristics

**Fig. 2:**
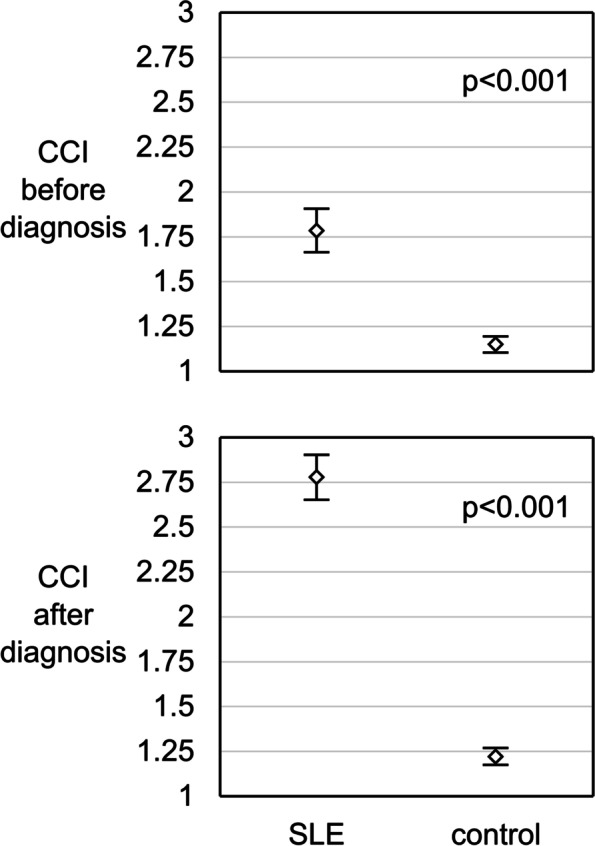
Age-adjusted CCI of SLE patients and matched controls prior to and after the index period. Bars denote 95% confidence intervals for the mean CCI of the group. Abbreviations: CCI, Charlson Comorbidity Index; SLE, systemic lupus erythematosus

A total of 1051 patients with SLE were matched with 5255 controls. Median age of the patients was 45.9 years and majority of the patients were females (85.1%). The post-index prevalence of depression, anxiety, and depression or anxiety in patients with SLE was 9.9%, 12.7%, and 17.8%, respectively (Table 1). The CCI of patients with SLE was significantly greater than the matched controls both before and after the index period (*p*<0.001; Fig. 2).

**Table 1:** Patient population characteristics

Parameter	Total SLE population	SLE patients with			Matched control population	Matched control with		
		Depression	Anxiety	Depression/anxiety		Depression	Anxiety	Depression/anxiety
***N*(%)**	1051 (100%)	104 (9.9%)	134 (12.7%)	187 (17.8%)	5255 (100%)	241 (4.6%)	307 (5.8%)	451 (8.6%)
**Female,*n*(%)**	894 (85.1%)	94 (90.4%)	123 (91.8%)	170 (90.9%)	4472 (85.1%)	218 (90.5%)	283 (92.2%)	411 (91.1%)
**Median age at diagnosis date (years)**	45.9	55.6	50.7	53.1	45.9	55.6	54.7	55.2
**Median CCI (SD) within 2 years**								
Prior to index period	1.8 (2.0)	2.8 (2.2)	2.4 (2.1)	2.4 (2.1)	1.2 (1.6)	1.8 (1.8)	1.8 (1.9)	1.9 (1.9)
Post-index period	2.8 (2.0)	3.9 (2.2)	3.4 (2.1)	3.5 (2.2)	1.2 (1.7)	1.9 (1.8)	2.0 (2.0)	2.0 (2.0)
**Death, n (%)**	86 (8.2%)	11 (10.6%)	12 (9.0%)	17 (9.1%)	210 (4.0%)	20 (8.3%)	15 (4.9%)	29 (6.4%)

Patient with depression, anxiety, depression/anxiety refers to patients (and controls) who have these mental comorbidities in the post-index period

*Abbreviations*: *CCI* Charlson Comorbidity Index, *SD* standard deviation, *SLE* systemic lupus erythematosus

Rate of patients who were prescribed systemic corticosteroids within 2 years before their SLE diagnosis date was (56.6%); for whom the median time from treatment start to diagnosis was 13 months. After that, steroid use within 2 years after SLE diagnosis was recorded for 85.2% of patients, and 98.6% of the latter remained on steroids for at least one year. Other agents used within 2 years after diagnosis included hydroxychloroquine (46.4%), azathioprine (35.0%), and biologics (5.8%) with rituximab being the most frequently prescribed biologic. Detailed treatment pattern analysis could not be performed as data from inpatient care (e.g., on cyclophosphamide use and intravenous steroids) were unavailable in the claims data.

Based on the steroid use during the index period of 6 months, 19.8% of patients did not receive steroids and 19.0% received high-dose steroids (Table 2).

**Table 2:** Steroid dosage of patients with SLE during the index period

Parameter	Steroid dosage^a^			
	0 mg/day	0–7.5 mg/day	7.5–20 mg/day	>20 mg/day
**N (%)**	208 (19.8%)	325 (30.9%)	318 (30.3%)	200 (19.0%)
**Female,*n*(%)**	186 (89.4%)	280 (86.2%)	270 (89.4%)	158 (79.0%)
**Median age at diagnosis date (years)**	46.5	51.9	45.3	39.2
**Mean CCI (SD) within 2 years**				
Prior to index period	1.6 (1.8)	2.1 (2.1)	1.8 (2.0)	1.5 (1.8)
Post-index period	2.7 (2.0)	3.0 (2.2)	2.7 (2.1)	2.5 (1.7)
**Death during follow-up, n (%)**	15 (7.2%)	26 (8.0%)	32 (10.1%)	13 (6.5%)

*Abbreviations*: *CCI* Charlson Comorbidity Index, *SD* standard deviation, *SLE* systemic lupus erythematosus

^a^Prednisone equivalent daily dose of steroid within 6 months post-SLE diagnosis date

### Risk of comorbidity in SLE

**Fig. 3:**
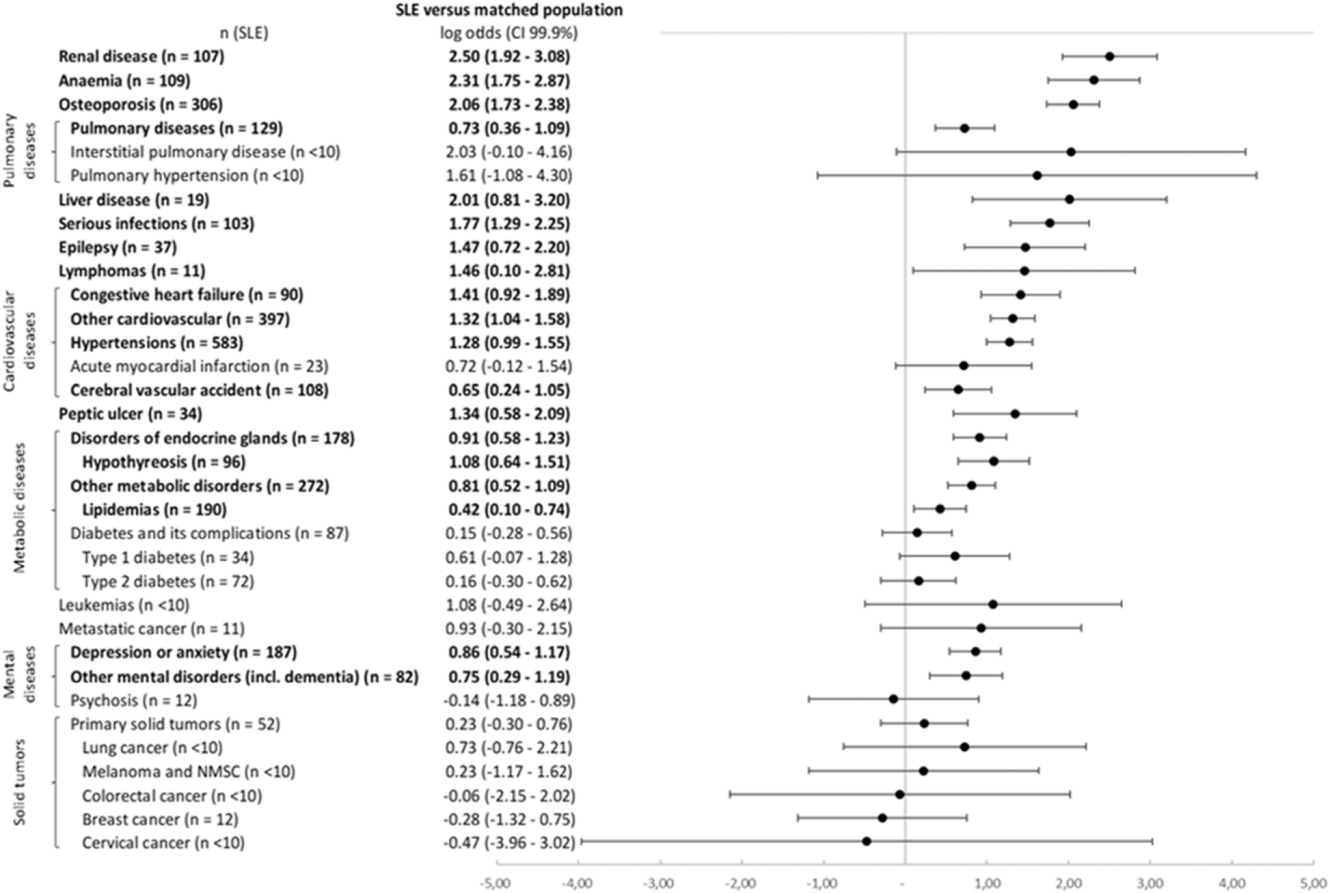
Log odds ratio of presenting comorbidities within 2 years post-index—SLE patients vs. matched population. Matching is based on age, gender, and region. Abbreviations: CI, confidence interval; SLE, systemic lupus erythematosus

Compared with matched controls, patients with SLE in the 2 years post-index period had a significantly greater risk of renal disease (OR 12.19 [99.9% CI 6.83–21.76]), anemia (OR 10.10 [99.9% CI 5.76–17.70]), osteoporosis (OR 7.82 [99.9% CI 5.64–10.84]), pulmonary diseases (OR 2.07 [99.9% CI 1.44–2.99]), liver disease (OR 7.47 [99.9% CI 2.27–24.59]), serious infections (OR 5.89 [99.9% CI 3.63–9.54]), epilepsy (OR 4.33 [99.9% CI 2.06–9.11]), lymphomas (OR 4.30 [99.9% CI 1.11–16.70]), congestive heart failure (OR 4.10 [99.9% CI 2.52–6.67]), other cardiovascular diseases (OR 3.74 [99.9% CI 2.86–4.89]), hypertension (OR 3.59 [99.9% CI 2.72–4.75]), cerebral vascular accident (OR 1.91 [99.9% CI 1.27–2.88]), peptic ulcer (OR 3.83 [99.9% CI 1.80–8.15]), endocrine gland disorders (OR 2.49 [99.9% CI 1.80–3.44]), hypothyroidism (OR 2.95 [99.9% CI 1.91–4.57]), other metabolic disorders (OR 2.25 [99.9% CI 1.68–3.00]), lipidaemia (OR 1.52 [99.9% CI 1.11–2.10]), depression or anxiety (OR 2.36 [99.9% CI 1.72–3.23]), and other mental disorders including dementia (OR 2.11 [99.9% CI 1.35–3.30]) (Supplementary Table S4). Figure 3 represents the log odds ratio data.

In most cases (including anxiety and depression), we found no significant difference across any of the 4 strata of steroid treatments on the risk of comorbidities, except for anemia and renal disease. The group with no steroid treatment served as the reference group. Patients with SLE who received >20 mg/day steroid treatment during the index period had a significantly greater risk of presence of anemia (OR 2.83 [99.9% CI 1.09–7.35]) and renal disease (OR 3.54 [99.9% CI 1.38–9.06]) in the 2-year post-index period. Patients on steroid doses of 0–7.5 mg/day and 7.5–20 mg/day did not have a significantly greater risk of these comorbidities (Supplementary Fig. S1).

### Risk of presenting depression, anxiety, or both

SLE patients and controls with depression or anxiety in the post-index period had greater CCI scores calculated before the index period compared with those who did not have comorbid depression or anxiety (*p*<0.001 for both; Supplementary Fig. S2).

**Fig. 4:**
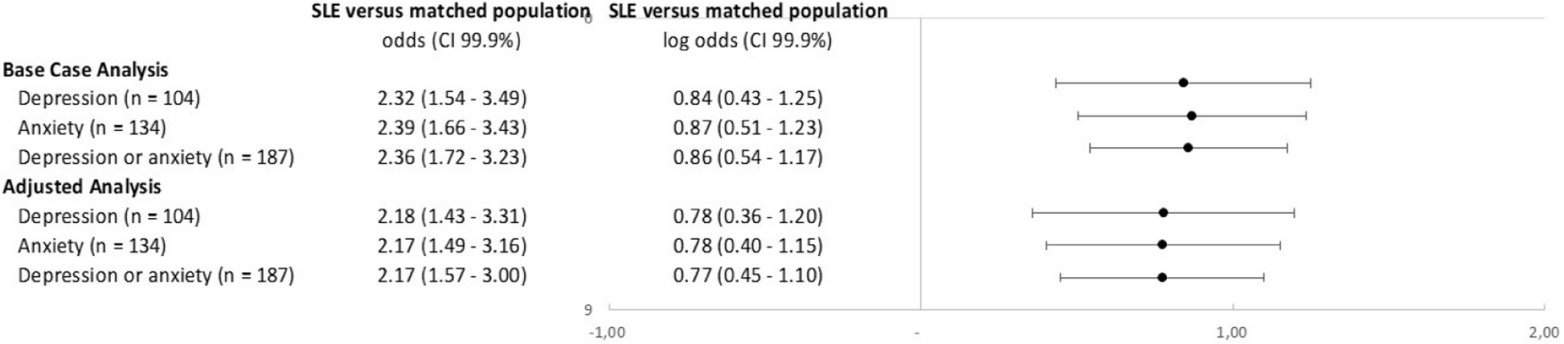
Odds ratio of presenting depression/anxiety within 2 years post-index—SLE patients vs. matched population. In the Base Case Analysis, the model was adjusted for age and gender. In the Adjusted Analysis, the model was additionally adjusted for CCI within 2 years prior to the diagnosis date. Matching is based on age, gender, and region. Abbreviations: CI, confidence interval; SLE, systemic lupus erythematosus

For the Base Case Analysis as well as Adjusted Analysis, patients with SLE had an approximately twofold greater chance of presenting the depression (Base Case Analysis, OR 2.32 [99.9% CI 1.54–3.49]; adjusted analysis, OR 2.18 [99.9% CI 1.43–3.31]), anxiety (Base Case Analysis, OR 2.39 [99.9% CI 1.66–3.43]; adjusted analysis, OR 2.17 [99.9% CI 1.49–3.16]), or both (Base Case Analysis, OR 2.36 [99.9% CI 1.72–3.23]; adjusted analysis, OR 2.17 [99.9% CI 1.57–3.00]) in the post-index period, than the general population (Fig. 4).

## Discussion

Patient register data clearly indicate that comorbidities have a crucial role in determining the HRQoL, ability to work, and mortality of patients with SLE [24]. Our present data confirm and extend these observations. In addition to existing population-based studies assessing the comorbidities associated with SLE burden [24–33], this is the first nationwide full-populational database study in Europe with 1:5 randomly matched controls from the general population that focuses on comorbidities in SLE, especially depression and anxiety.

The prevalence of comorbid depression and anxiety in patients with SLE in the administrative claims data analyzed in our study was 9.9% and 12.7%, respectively, which is lower than the self-reported or healthcare professional-reported prevalence found by a recent systematic review and meta-analysis (depression - 35.0% [95% CI 29.9–40.3%]) and anxiety - 25.8% [95% CI 19.2–32.9%]) [17]. Our results are comparable with a full population study conducted in Crete that reported the prevalence of depression and anxiety to be 26.7% and 10.7%, respectively [34].

Patients with SLE in our study had greater chance of presenting the lymphomas, endocrine gland disorders, metabolic disorders, mental disorders, cardiovascular diseases, pulmonary diseases, renal disease, liver disease, and infections during the post-index periods than the matched controls. This comorbidity profile is comparable with published European data. A recent Finnish study analyzed data of patients with SLE and matched controls from 2000 to 2015 and reported comorbid cardiovascular diseases (33%), malignancies (27%), and neurological diseases (10%) present at diagnosis to be the leading causes of death [35]. A full population study in Crete also reported a high prevalence of comorbid thyroid disease (45.6%), mental disorders (42.1%), dyslipidemia (33.3%), and hypertension (24.6%) [34].

In our study, patients with SLE receiving a high steroid dose (>20 mg/day) had a greater risk of having anemia or renal disease compared with matched controls. It can mean that the severity of the disease was greater in these patients resulting in higher steroid use and therefore worse renal disease and anemia, but also that steroids may have been used to treat these. We found no difference in the risk of any of the other comorbidities among patients with different steroid doses. Although other studies have examined comorbidities as a function of steroid dose [36–39], the observed comorbidity profiles were not the same either due to assessment of specific comorbidities [36–38] or varying study population [39].

Comorbidities such as cardiovascular diseases [40], depression, and anxiety [19, 41], in patients with SLE, significantly impair HRQoL. The latter two are also involved with body image and fatigue severity, which further affect HRQoL [20]. Moreover, depression and anxiety can indicate increased inflammatory activity, associated with SLE flares [42, 43]. In our study, patients with SLE had a twofold higher chance of presenting the depression, anxiety, or both compared with matched controls in both the Base Case Analysis and the Adjusted Analysis, thus highlighting the burden of SLE. Inadequate control at an early stage may lead to further deterioration in physical, mental, and social functioning in these patients. Hence, optimization of pharmacological and non-pharmacological treatments to alleviate depression or anxiety-related symptoms could positively impact HRQoL [41].

Infections and cardiovascular diseases are reported to be the leading causes of death in SLE [44–46]. In the current study too, the presence of serious infections was sixfold greater in patients with SLE compared with matched controls after the diagnosis date. This is consistent with evidence suggesting that infections have a large effect in accelerated mortality in patients with SLE [1]. As skin-, urinary-, and lower respiratory tract-related infections are known to be common in patients with SLE [47, 48], early identification and adequate treatment of infection-related symptoms appear to be essential for reducing mortality in patients with SLE.

In line with recent European guidelines [49, 50] that recommend the use of biologics in the second or subsequent line for SLE treatment, majority of the patients in our study were on corticosteroids, with only 5.8% on biologics. However, treatment pattern analysis was hampered due to a lack of reliable information on use of medications during hospitalization in the claims dataset. Although we used a conservative approach to estimate the diagnosis date (e.g., two newly recorded SLE ICD codes + flare indicated by immediate treatment), we observed that 58% of the patients received some form of SLE relevant treatment via pharmacy prescriptions before the date of SLE diagnosis appeared in the dataset, indicating that a not differentiated autoimmune disease state may often precede the diagnosis.

The main advantage of this study is the full population analysis which enables obtaining deep insights into the burden of SLE. Limited evidence exists on the complete comorbidity profile in patients with SLE with an emphasis on depression and anxiety. This study bridges the knowledge gap by analyzing patients and matched controls in a 1:5 ratio to obtain insights.

There are some limitations of this study. The data were collected for health insurance reimbursement claims purposes and not for research. Data on the therapy used during hospitalization of patients with SLE (i.e., treatment with cyclophosphamide, other immunosuppressive therapies, or use of glucocorticoids) are unavailable. Hence, we cannot accurately determine the exact disease activity and severity and treatment patterns. No data was available on clinical details such as disease phenotype, disease severity, or patient-reported outcomes either. All comorbidities were identified through claims data; however, misdiagnosis might be possible. Some diagnoses may be present on prescriptions to enable the prescription of certain medications. This is especially true for depression and anxiety. Due to data handling regulations, we could not report patient-level data or aggregate data obtained from less than 10 patients (including patient numbers). This study utilizes claims data from Hungary, and findings may not be generalizable to other populations. When analyzing the effect of steroid dose on comorbidities, the amount of steroid prescribed may be influenced by comorbidities which were present pre-index which can cause confounding. this was not adjusted for in the analysis. Adjusting for the total CCI score in the final set of analyses may mask the influence of specific comorbidities.

We observed that most comorbidities have a higher risk of being present in patients with SLE than in matched controls. The greatest risk increase was observed in renal disease, anemia, and osteoporosis. We also observed that depression and anxiety have a higher risk of being present in patients with SLE after controlling for the presence of other comorbidities, suggesting that depression and anxiety, at least partly, are not caused by the higher comorbid nature of SLE patients. Overall, the enormity of comorbidities specifically depression and anxiety emphasize the importance of early diagnosis and intervention incorporating attention to comorbidities in SLE patients.

## Supplementary Information

**Additional file 1: Supplementary Table S1**: Treatments for systemic lupus erythematosus. Abbreviation: ATC – Anatomical Therapeutic Chemical.

**Additional file 2: Supplementary Table S2**: ICD-10 codes for comorbidities. Abbreviations: ICD-10 – International Classification of Diseases, 10^th^ edition.

**Additional file 3: Supplementary Table S3**: Calculation of CCI.

**Additional file 4: Supplementary Table S4**: Risk of comorbidity in SLE patients within 2 years prior diagnosis date and within the 2 years post-index period.

**Additional file 5: Supplementary Figure S1**: Odds ratio of presenting comorbidities within 2 years post-index period for patients with SLE who received different doses of steroids vs. those who did not receive steroids in the index period.

**Additional file 6: Supplementary Figure S2**: Comparison of CCI prior to diagnosis date of individuals presenting depression or anxiety in the post-index period, within SLE patients and matched controls.

## Data Availability

The dataset that was used is held by the National Health Insurance Fund (NHIF) of Hungary (http://www.neak.gov.hu, e-mail: neak@neak.gov.hu). Access to the individual-level data is available after filing a formal data access request with adatkeres@neak.gov.hu. Requestors need to accept the terms and conditions of the data request and may need to pay the corresponding data access fee. The terms of the contract for data access do not allow the reporting of any data of a single individual or results which come from aggregating the data of <10 individuals. Therefore, a de-identified dataset could not be provided. Taking these requirements into consideration, the results can be published. A supplementary dataset was created which contains the patient counts derived from the original data. This is a retrospective secondary database study, no new data were generated or analyzed in support of this research.
