# Supplementary material for: High risk of depression, anxiety, and an unfavorable complex comorbidity profile is associated with SLE: a nationwide patient-level study
Source: Arthritis Res Ther. 2022 May 19;24:116. doi: 10.1186/s13075-022-02799-6 (PMC9118724; doi:10.1186/s13075-022-02799-6)
Supplement: Supplementary file 1 — Additional file 1: Supplementary Table S1: Treatments for systemic lupus erythematosus. Abbreviation: ATC – Anatomical Therapeutic Chemical. [file 13075_2022_2799_MOESM1_ESM.docx]

**Supplementary Table S1: Treatments for systemic lupus erythematosus**

| **Treatment** | **ATC code** |
| --- | --- |
| Hydroxychloroquine | P01BA01 |
| Corticosteroids for systemic use | H02 |
| Mycophenolate Mofetil | L04AA06 |
| Cyclosporine | L04AD01 |
| Azathioprine | L04AX01 |
| Rituximab | L01XC02 |
| Adalimumab | L04AB04 |
| Tocilizumab | L04AC07 |
| Etanercept | L04AB01 |
| Infliximab | L04AB02 |
| Alteplase | B01AD02 |
| Certolizumab | L04AB05 |
| Abatacept | L04AA24 |
