# Supplementary material for: High risk of depression, anxiety, and an unfavorable complex comorbidity profile is associated with SLE: a nationwide patient-level study
Source: Arthritis Res Ther. 2022 May 19;24:116. doi: 10.1186/s13075-022-02799-6 (PMC9118724; doi:10.1186/s13075-022-02799-6)
Supplement: Supplementary file 2 — Additional file 2: Supplementary Table S2: ICD-10 codes for comorbidities. Abbreviations: ICD-10 – International Classification of Diseases, 10th edition. [file 13075_2022_2799_MOESM2_ESM.docx]

**Supplementary Table S2: ICD-10 codes for comorbidities**

| **Comorbidity** | **ICD-10 codes** |
| --- | --- |
| **Malignancies** | C00, C01, C02, C03, C04, C05, C06, C07, C08, C09, C10, C11, C12, C13, C14, C15, C16, C17, C19, C20, C21, C22, C23, C24, C25, C26, C30, C31, C32, C33, C37, C38, C39, C40, C41, C45, C46, C47, C48, C49, C51, C52, C54, C55, C56, C57, C58, C60, C61, C62, C63, C64, C65, C66, C67, C68, C69, C70, C71, C72, C73, C74, C75, C76, C77, C78, C79, C80, C81, C82, C83, C84, C85, C88, C90, C91, C92, C93, C94, C95, C96, C97, D00, D01, D02, D06, D07, D09, C50, D05, C53, C18, D01, C34, C43, C44, D03, D04 |
| Primary solid tumors | C00, C01, C02, C03, C04, C05, C06, C07, C08, C09, C10, C11, C12, C13, C14, C15, C16, C17, C19, C20, C21, C22, C23, C24, C25, C26, C30, C31, C32, C33, C37, C38, C39, C40, C41, C45, C46, C47, C48, C49, C51, C52, C54, C55, C56, C57, C58, C60, C61, C62, C63, C64, C65, C66, C67, C68, C69, C70, C71, C72, C73, C74, C75, C76, C97, D00, D01. D02, D06, D07, D09 |
| Colorectal cancer | C18, D01 |
| Lung cancer | C34 |
| Melanoma and NMSC | C43, C44, D03, D04 |
| Breast cancer | C50; D05 |
| Cervical cancer | C53 |
| Lymphomas | C81, C82, C83, C84, C85, C88, C90 |
| Myeloma multiplex | C90 |
| Leukemias | C91, C92, C93, C94, C95, C96 |
| Metastatic cancer | C77, C78, C79, C80 |
| **Metabolic disorders** | E70, E71, E72, E73, E74, E75, E76, E77, E78, E79, E80, E83, E84, E85, E86, E87, E88, E89, E90 |
| Lipidemia | E78 |
| Disorders of the endocrine glands | E00, E01, E02, E03, E04, E05, E06, E07, E20, E21, E22, E23, E24, E25, E26, E27, E28, E29, E30, E31, E32, E34, E35 |
| Hypothyroidism | E03 |
| Diabetes | E10; E11; E13; E14 |
| Type 1 diabetes | E10 |
| Type 2 diabetes | E11 |
| **Mental disorders** |  |
| Depression | F31; F32; F33 |
| Anxiety | F41 |
| Psychosis | F20, F21, F22, F23, F24, F25, F28, F29 |
| Other mental disorders | F00, F01, F02, F03, F04, F05, F06, F07, F09, F10, F11, F12, F13, F14, F15, F16, F17, F18, F19, F30, F34, F38, F39, F40, F42, F43, F44, F45, F48, F50, F51, F52, F53, F54, F55, F59, F60, F61, F62, F63, F64, F65, F66, F68, F69, F70, F71, F72, F73, F78, F79, F80, F81, F82, F83, F84, F88, F89, F90, F91, F92, F93, F94, F95, F98, F99 |
| **Cardiovascular and cerebrovascular disorders** |  |
| Cerebral vascular accident | G45, G46, I60, I61, I62, I63, I64, I65, I66, I67, I68, I69 |
| Acute myocardial infarction | I21, I22, I23, I25 |
| Congestive heart failure | I50 |
| Hypertensions | I10, I11, I12, I13, I15 |
| Other cardiovascular disorders | I00, I01, I02, I05, I06, I07, I08, I09, I20, I24, I25, I26, I27, I28, I30, I31, I32, I33, I34, I35, I36, I37, I38, I39, I40, I41, I42, I43, I44, I45, I46, I47, I48, I49, I51, I52, I70, I71, I72, I73, I74, I77, I78, I79, I80, I81, I82, I83, I84, I85, I86, I87, I88, I89, I95, I97, I98, I99 |
| **Pulmonary** **diseases** | I27, J40, J41, J42, J43, J44, J45, J46, J47, J60, J61, J62, J63, J64, J65, J66, J67, J84 |
| Interstitial pulmonary disease | J84 |
| Pulmonary hypertension | I27 |
| **Anemia** | D60, D61, D62, D63, D64 |
| **Epilepsy** | G40, G41 |
| **Liver disease** | K70, K71, K73, K74 |
| **Osteoporosis** | M80, M81, M82, M83, M84, M85 |
| **Peptic ulcer** | K25, K26, K27, K28 |
| **Renal disease** | N01, N03, N05, N07, N18, N19, N25 |
| **Serious infections (inpatient only)** | A00-A09, A15-A28, A30-A44, A46, A48, A49, A52-A55, A57-A60, A65-A71, A74, A75, A77-A96, A98, A99, B00-B09, B15-B27, B30, B33-B49, B50-B60, B64-B83, B85-B92, B94-B97, B99, H10, H60, H65, H66, J00-J04, J06, J10-J18, J20, J21, J30-J32, J37, J39-J42, K05, K12, K20, K29, K81, L04, L08, L20, L21, L23, L24, L27, L30, L98, M65, N00, N01, N03-N05, N11-N12, N30, N45, N72, N76, R23 |

**Abbreviations:** ICD-10 – International Classification of Diseases, 10^th^ edition
