# Supplementary material for: High risk of depression, anxiety, and an unfavorable complex comorbidity profile is associated with SLE: a nationwide patient-level study
Source: Arthritis Res Ther. 2022 May 19;24:116. doi: 10.1186/s13075-022-02799-6 (PMC9118724; doi:10.1186/s13075-022-02799-6)
Supplement: Supplementary file 3 — Additional file 3: Supplementary Table S3: Calculation of CCI. [file 13075_2022_2799_MOESM3_ESM.docx]

**Supplementary Table S3: Calculation of CCI**

| **Comorbidity** | **Score** |
| --- | --- |
| Acute myocardial infarction | 1 |
| Cerebral vascular accident | 1 |
| Congestive heart failure | 1 |
| Connective tissue disorder | 1 |
| Dementia | 1 |
| Diabetes | 1 |
| Liver disease | 1 |
| Peptic ulcer | 1 |
| Peripheral vascular disease | 1 |
| Pulmonary diseases | 1 |
| Malignancies | 2 |
| Diabetes complications | 2 |
| Paraplegia | 2 |
| Renal disease | 2 |
| Metastatic cancer | 3 |
| Severe liver disease | 3 |
| HIV | 6 |
| **Age** |  |
| 50 ≤ age < 60 | 1 |
| 60 ≤ age < 70 | 2 |
| 70 ≤ age < 80 | 3 |
| 80 ≤ age | 4 |
