# Supplementary material for: High risk of depression, anxiety, and an unfavorable complex comorbidity profile is associated with SLE: a nationwide patient-level study
Source: Arthritis Res Ther. 2022 May 19;24:116. doi: 10.1186/s13075-022-02799-6 (PMC9118724; doi:10.1186/s13075-022-02799-6)
Supplement: Supplementary file 4 — Additional file 4: Supplementary Table S4: Risk of comorbidity in SLE patients within 2 years prior diagnosis date and within the 2 years post-index period. [file 13075_2022_2799_MOESM4_ESM.docx]

**Supplementary Table S4: Risk of comorbidity in SLE patients within 2 years prior diagnosis date and within the 2 years post-index period**

| **Comorbidities** | | **2 years prior diagnosis date** | | | **2 years post-index period** | | |
| --- | --- | --- | --- | --- | --- | --- | --- |
|  |  | **SLE, n (%)**  **N=1,051** | **Controls, n (%) N=5,255** | **OR (99.9% CI)** | **SLE, n (%)**  **N=1,051** | **Controls, n (%) N=5,255** | **OR (99.9% CI)** |
| **Malignancies** | **Primary solid tumors** | 34 (3.2%) | <10 | 1.00 (0.53–1.90) | 52 (4.9%) | 210 (4.0%) | 1.26 (0.74–2.16) |
|  | Colorectal cancer | <10 | 170 (3.2%) | 2.17 (0.22–21.46) | <10 | 16 (0.3%) | 0.94 (0.12–7.54) |
|  | Lung cancer | <10 | <10 | 0.71 (0.02–24.33) | <10 | 17 (0.3%) | 2.08 (0.47–9.18) |
|  | Melanoma and NMSC | <10 | <10 | 1.44 (0.38–5.47) | <10 | 28 (0.5%) | 1.26 (0.31–5.10) |
|  | Breast cancer | 10 (1.0%) | 28 (0.5%) | 0.70 (0.23–2.15) | 12 (1.1%) | 79 (1.5%) | 0.75 (0.27–2.12) |
|  | Cervical cancer | <10 | 71 (1.4%) | 1.02 (0–NA) | <10 | <10 | 0.62 (0.02–20.57) |
|  | **Lymphomas** | <10 | <10 | 9.25 (2.50–34.14) | 11 (1.0%) | 13 (0.2%) | 4.30 (1.11–16.70) |
|  | **Leukemia** | <10 | <10 | 3.37 (0.40–28.52) | <10 | 12 (0.2%) | 2.94 (0.61–14.15) |
|  | **Metastatic cancer** | <10 | <10 | 1.48 (0.27–7.94) | 11 (1.0%) | 22 (0.4%) | 2.53 (0.74–8.62) |
| **Metabolic diseases** | **Disorders of endocrine glands** | 150 (14.3%) | <10 | 2.54 (1.79–3.60) | 178 (16.9%) | 404 (7.7%) | 2.49 (1.80–3.44) |
|  | Hypothyroidism | 73 (6.9%) | 329 (6.3%) | 2.68 (1.64–4.37) | 96 (9.1%) | 175 (3.3%) | 2.95 (1.91–4.57) |
|  | **Diabetes and its complications** | 73 (6.9%) | 144 (2.7%) | 1.10 (0.70–1.74) | 87 (8.3%) | 384 (7.3%) | 1.16 (0.76–1.76) |
|  | Type 1 diabetes | 20 (1.9%) | 336 (6.4%) | 1.20 (0.52–2.75) | 34 (3.2%) | 95 (1.8%) | 1.83 (0.94–3.60) |
|  | Type 2 diabetes | 57 (5.4%) | 84 (1.6%) | 1.04 (0.63–1.73) | 72 (6.9%) | 312 (5.9%) | 1.18 (0.74–1.86) |
|  | **Other metabolic disorders** | 231 (22.0%) | 275 (5.2%) | 1.90 (1.40–2.59) | 272 (25.9%) | 779 (14.8%) | 2.25 (1.68–3.00) |
|  | Lipidaemia | <10 | 745 (14.2%) | 1.43 (1.02–2.01) | 190 (18.1%) | 699 (13.3%) | 1.52 (1.11–2.10) |
| **Mental diseases** | **Depression or anxiety** | 88 (8.4%) | <10 | 1.59 (1.12–2.25) | 187 (17.8%) | 451 (8.6%) | 2.36 (1.72–3.23) |
|  | Psychosis | 11 (1.0%) | 295 (5.6%) | 0.86 (0.29–2.53) | 12 (1.1%) | 69 (1.3%) | 0.87 (0.31–2.45) |
|  | **Other mental disorders** | <10 | 64 (1.2%) | 1.73 (1.05–2.83) | 82 (7.8%) | 204 (3.9%) | 2.11 (1.35–3.30) |
| **Cardiovascular diseases** | **Cerebral vascular accident** | 92 (8.8%) | <10 | 1.77 (1.14–2.74) | 108 (10.3%) | 315 (6.0%) | 1.91 (1.27–2.88) |
|  | **Acute MI** | 26 (2.5%) | 287 (5.5%) | 2.41 (1.08–5.36) | 23 (2.2%) | 58 (1.1%) | 2.05 (0.89–4.71) |
|  | **CHF** | 47 (4.5%) | 56 (1.1%) | 2.74 (1.47–5.11) | 90 (8.6%) | 127 (2.4%) | 4.10 (2.52–6.67) |
|  | **Hypertension** | 470 (44.7%) | 94 (1.8%) | 2.33 (1.74–3.10) | 583 (55.5%) | 1795 (34.2%) | 3.59 (2.72– 4.75) |
|  | **Other cardiovascular diseases** | 386 (36.7%) | 1684 (32.0%) | 3.93 (2.99–5.17) | 397 (37.8%) | 867 (16.5%) | 3.74 (2.86– 4.89) |
| **Pulmonary diseases** | **Pulmonary diseases** | 134 () | 810 (15.4%) | 2.35 (1.63–3.39) | 129 (12.3%) | 338 (6.4%) | 2.07 (1.44–2.99) |
|  | Interstitial pulmonary disease | <10 | 313 (6.0%) | 10.13 (1.34–76.44) | <10 | <10 | 7.62 (0.90–64.40) |
|  | Pulmonary hypertension | 191 (18.2%) | <10 | 2.51 (0.04– 142.28) | <10 | <10 | 5.02 (0.34– 73.88) |
| **Others** | **Anemia** | 102 (9.7%) | 55 (1.0%) | 10.82 (6.10–19.20) | 102 (9.7%) | 56 (1.1%) | 10.10 (5.76–17.70) |
|  | **Epilepsy** | 23 (2.2%) | 35 (0.7%) | 2.78 (1.17–6.58) | 37 (3.5%) | 44 (0.8%) | 4.33 (2.06–9.11) |
|  | **Liver disease** | 20 (1.9%) | 25 (0.5%) | 5.69 (1.94–16.72) | 19 (1.8%) | 13 (0.2%) | 7.47 (2.27–24.59) |
|  | **Osteoporosis** | 138 (13.1%) | 18 (0.3%) | 3.29 (2.20–4.93) | 306 (29.1%) | 347 (6.6%) | 7.82 (5.64–10.84) |
|  | **Peptic ulcer** | 108 (10.3%) | 276 (5.3%) | 2.67 (1.21–5.92) | 34 (3.2%) | 46 (0.9%) | 3.83 (1.80–8.15) |
|  | **Renal disease** | 79 (7.5%) | 42 (0.8%) | 17.82 (8.25–38.52) | 107 (10.2%) | 51 (1.0%) | 12.19 (6.83–21.76) |
|  | **Serious infections** | 152 (14.5%) | 52 (1.0%) | 10.30 (6.49–16.34) | 103 (9.8%) | 96 (1.8%) | 5.89 (3.63–9.54) |

Each patient with SLE was matched by age, gender and region to 5 patients from the general population, and odds ratios are calculated with a logistic regression model. Patient numbers lower than 10 are not presented**.**

**Abbreviations:** CHF – Congestive Heart Failure, CI – Confidence Interval, MI – Myocardial Infarction, NMSC – Non-melanoma Skin Cancer, OR – Odds Ratio, SLE – Systemic Lupus Erythematosus
