# Supplementary figures and images for: High risk of depression, anxiety, and an unfavorable complex comorbidity profile is associated with SLE: a nationwide patient-level study
Source: Arthritis Res Ther. 2022 May 19;24:116. doi: 10.1186/s13075-022-02799-6 (PMC9118724; doi:10.1186/s13075-022-02799-6)

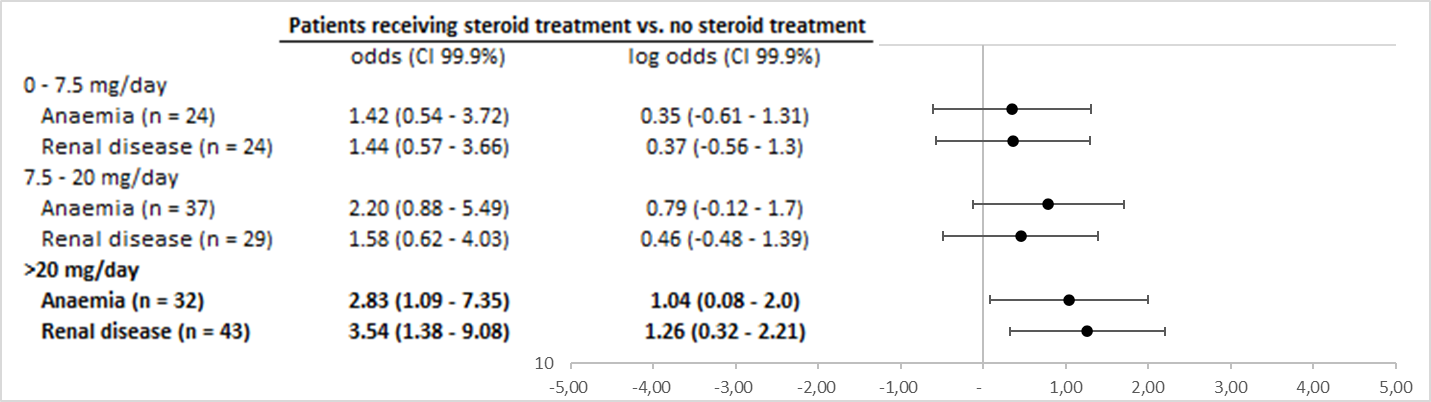

Supplement: Supplementary file 5 — Additional file 5: Supplementary Figure S1: Odds ratio of presenting comorbidities within 2 years post-index period for patients with SLE who received different doses of steroids vs. those who did not receive steroids in the index period. [file 13075_2022_2799_MOESM5_ESM.docx]

**
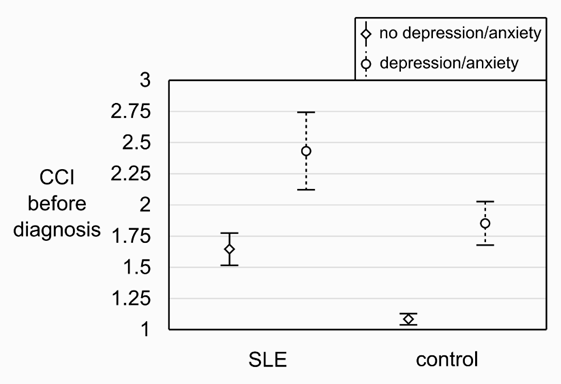
**

Supplement: Supplementary file 6 — Additional file 6: Supplementary Figure S2: Comparison of CCI prior to diagnosis date of individuals presenting depression or anxiety in the post-index period, within SLE patients and matched controls. [file 13075_2022_2799_MOESM6_ESM.docx]
